# Contrast-Enhanced CT-Based Radiomics Nomogram for Prediction of Pathologic T3a Upstaging in Clinical T1 RCC

**DOI:** 10.3390/diagnostics15040443

**Published:** 2025-02-12

**Authors:** Di Yin, Keruo Wang, Hongyi Xu, Yunfei Guo, Baoxin Qian, Dengyi Duan, Yiming Li, Wenyi Zhang, Zhengyang Li, Yang Zhao

**Affiliations:** 1 Department of Radiology, The Second Hospital of Tianjin Medical University, Tianjin 300211, China; 18109714901@163.com (D.Y.); xuhongyitmu@163.com (H.X.); gelly52100@163.com (Y.G.); d18875255938@163.com (D.D.); 17853295805@163.com (Y.L.); zhangwenyilucky@126.com (W.Z.); zy849312853@163.com (Z.L.); 2 Tianjin Institute of Urology, The Second Hospital of Tianjin Medical University, Tianjin 300211, China; wangkr3616@tmu.edu.cn; 3 Huiying Medical Technology (Beijing), Beijing 100192, China; qianbaoxin@huiyihuiying.com

**Keywords:** renal cell carcinoma, radiomics, nomogram, pathologic upstaging

## Abstract

**Background/Objectives**: To develop a nomogram for the preoperative prediction of pathologic T3a (pT3a) upstaging in patients with clinical T1(cT1) renal cell carcinoma (RCC). **Methods**: A total of 169 cT1 patients with RCC with preoperative contrast-enhanced CT (CECT) and clinical data were enrolled in this study. Afterwards, the sample was split randomly into training and testing sets in a 7:3 ratio. Radiomics features were extracted and selected from the whole primary tumor on CECT images to develop radiomics signatures. The nomogram was constructed using the obtained radiomics signature and clinical risk factors. The predictive performance of different models was evaluated and visualized using receiver operator characteristic (ROC) curves. **Results**: In total, 26 radiomics features were selected for the radiomics signature construction. The radiomics signature yielded area under the curve (AUC) values of 0.945 and 0.873 in the training and testing sets, respectively. The nomogram integrating radiomics signature and predictive clinical factors, including tumor size and neutrophil–lymphocyte ratio (NLR), achieved higher predictive performance in the training [AUC, 0.958; 95% confidence interval (CI): 0.921, 0.995] and testing (AUC, 0.913; 95% CI: 0.814, 1.000) sets. Good calibration was achieved for the nomogram in both the training and testing sets (Brier score = 0.082 and 0.098). Decision curve analysis (DCA) demonstrated that the nomogram was clinically useful in predicting pT3a upstaging, with a corresponding net benefit of 0.378. **Conclusions**: The proposed nomogram can preoperatively predict pT3a upstaging in cT1 RCC and serve as a non-invasive imaging marker to guide individualized treatment.

## 1. Introduction

Renal cell carcinoma (RCC) is a spectrum of cancers arising from the renal tubular epithelium, accounting for about 85% of all kidney malignancy among adults [1]. The incidence of RCC continued to climb by 2.8% per year, driven by the incidental detection of renal masses with the use of advanced imaging modalities [2]. Based on recommendations from the European Association of Urology (EAU) guidelines [3], partial nephrectomy (PN) is currently the most recommended surgery for localized T1 RCC, yet its efficacy for T3 RCC remains uncertain [4,5,6,7]. Early detection of RCC supports the use of PN as the preferred treatment due to its potential advantages in improving functional and cardiovascular outcomes. Moreover, PN surgery is also being explored for larger and more complex renal masses [8]. However, clinically localized RCC may ultimately be found to be a more aggressive disease on final pathology. It is reported that an estimated 5–14% cT1 patients with RCC confirmed occult pT3a upstaging after surgery, characterized by sinus fat, renal vein, collecting system, and perinephric fat invasion without inferior vena cava invasion [9]. Recent retrospective cohort studies have indicated that patients with cT1 renal cell carcinoma who are pathologically upstaged to T3a exhibit a poorer prognosis compared to those with pT1 disease [10,11,12,13]. Thus, pre-identifying patients who are at risk of pT3a upstaging can assist in preoperative staging and assigning individualized follow-up protocols.

Renal mass biopsy (RMB) is now the gold standard for the preoperative determination of the pathological patterns of RCC. However, issues of biopsy tract seeding and relevant complications [14] have arisen. Previous studies have attempted to investigate the correlation between clinical factors and the upstaging of cT1 RCC to pT3a, including age, RENAL score, and laboratory indices such as the serum aspartate aminotransferase (AST) to alanine aminotransferase (ALT) ratio (AST/ALT ratio) [13,15,16,17]. However, comprehensive collection of clinical data can be challenging and time-consuming.

Contrast-enhanced CT (CECT) is the most commonly used imaging modality for the preoperative characterization of RCC and postoperative monitoring of tumor progression. Qualitative CECT features, such as enhancement, intratumoral necrosis, and peritumoral neovascularity, provide indications of RCC staging, histology, and even response to therapy [18,19,20]. A previous investigation of CECT demonstrated a significant correlation between irregular tumor margins and pT3a upstaging in cT1 RCC [21]. However, these morphological characteristics, defined subjectively by radiologists, lack reproducibility and accuracy. And the visual assessment of subtle invasion into the renal vein or sinus fat can be challenging on traditional CT imaging. Therefore, there is an urgent need in clinical practice for quantitative and reliable tools with which to assess RCC objectively.

Radiomics is an emerging non-invasive tool that can extract and analyze quantitative features reflecting underlying tumors phenotype and heterogeneity from medical images, contributing greatly to clinical decision support [22,23]. Recent studies have been conducted for interpreting the association between radiomic features and biological behavioral patterns of RCC [24,25]. Previous studies have demonstrated that radiomics features can provide superior performance in the accurate diagnosis, tumor staging, pathology grading, and treatment response assessment of RCC [26,27,28]. However, reports involving the CECT radiomics approach for predicting pT3a upstaging from cT1 RCC are still rare.

The aim of this study was to develop and validate a nomogram integrating the radiomics signature with clinical risk factors to predict pT3a upstaging in cT1 patients with RCC, which aids in optimizing the pretreatment counseling of patients.

## 2. Materials and Methods

### 2.1. Study Population

**Figure 1:**
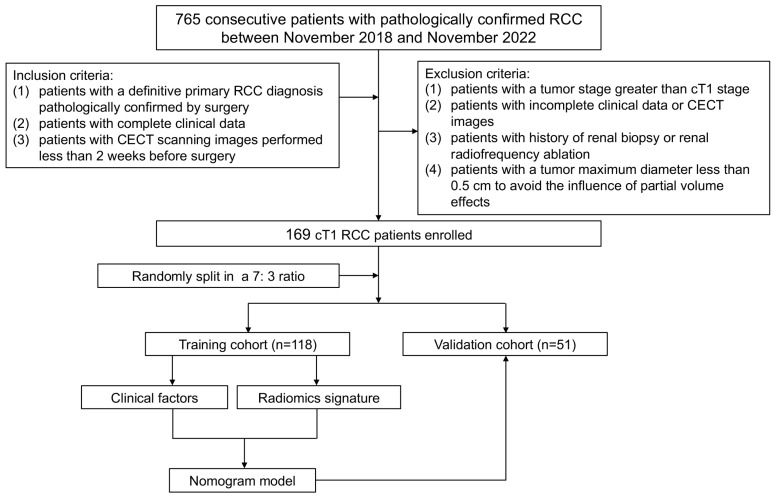
Overview of the workflow and patient recruitment in our study.

All patients signed informed consent forms for the use of surgically removed tissue for pathological and scientific research purposes before surgery. A total of 765 consecutive patients with pathologically confirmed RCC who underwent partial nephrectomy (PN) or radical nephrectomy (RN) at our hospital between November 2018 and November 2022 were retrospectively searched. The inclusion criteria entailed the following: (i) patients with a definitive primary RCC diagnosis pathologically confirmed by surgery; (ii) patients with complete clinical data; and (iii) patients with CECT scanning images performed less than 2 weeks before surgery. The exclusion criteria entailed the following: (i) patients with a tumor stage greater than cT1; (ii) patients with incomplete clinical data or CECT images; (iii) patients with a history of renal biopsy or renal radiofrequency ablation; and (iv) patients with a maximum tumor diameter of less than 0.5 cm to avoid the influence of partial volume effects. Finally, a total of 169 cT1 patients with RCC (126 males and 43 females; mean age: 61.17 ± 11.72 years) were enrolled in our study, consisting of the upstaging group (43 males and 12 females; mean age: 64.09 ± 11.35 years) and the non-upstaging group (83 males and 31 females; mean age: 59.76 ± 11.68 years). The whole dataset was divided randomly into a training (*n* = 118) and a testing set (*n* = 51) in a 7:3 ratio. An overall flowchart of this study is shown in Figure 1.

### 2.2. Clinical Data

Baseline clinical data, including sex, age, tumor size, RENAL score, body mass index (BMI), hemoglobin (Hb), neutrophil–lymphocyte ratio (NLR), albumin–globulin ratio (AGR), fibrinogen (FIB), and clinical and pathologic T stage were derived from our medical records. In this study, tumor size referred to the maximal tumor length measured on the preoperative CECT images. According to the 2017 American Joint Committee on Cancer (AJCC) staging system, category-T1 tumors are defined as tumors ≤ 7 cm confined within renal capsule; category-T3a tumors are defined as the invasion of the renal sinus, collecting system, perinephric fat, or renal vein, regardless tumor size [29]; and upstaging is defined as a final T3a pathology for preoperative cT1 patients with RCC.

### 2.3. CECT Image Acquisition

All CECT scanning were performed using 64-row multidetector CT scanners (Siemens SOMATOM Definition Edge, Munich, Germany). The scanning parameters were 120 kV, smart mAs, matrix of 512, 5 mm slice thickness, and a 5 mm interval. A dose of 1.5 mL/kg body weight iohexol was injected into the antecubital vein at a flow rate of 3.5 mL/s for the CECT images, followed by a bolus of a 20 mL saline flush. Corticomedullary phase (CMP), nephrographic phase (NP), and excretory phase (EP) were acquired at 28 s, 80 s, and 200 s after intravenous administration of contrast.

### 2.4. Tumor Segmentation and Radiomics Feature Extraction

Tumor segmentation and radiomics features extraction were performed in the RadCloud platform (Huiying Medical Technology Beijing Co., Ltd., Beijing, China, https://mics.huiyihuiying.com/ (accessed on 26 March 2024)). CMP, NP, and EP images, in DICOM format, were uploaded onto the RadCloud platform. The three-dimensional (3D) segmentation of tumors was performed by a radiologist (with 4 years’ experience in abdominal CT interpretation), who was blind to the clinical and pathologic data. Regions of interest (ROIs) were manually delineated, slice-by-slice, along the margin of tumors on axial CMP, NP, and EP images for each patient, with the top and bottom slices excluded to minimize the influence of partial volume effects. The final segmentation results were reviewed by a senior radiologist with 12-year experience in abdominal CT interpretation. Radiomics features were then extracted from the entire ROIs of each patient, and z-score normalization was applied to normalize the data. Finally, features with intra-class correlation coefficients (ICCs) greater than 0.80 were included in the subsequent analysis.

### 2.5. Feature Selection and Radiomics Signature Construction

To avoid over-fitting and reduce the dimensionality of radiomics features, the variance threshold, select K best, and least absolute shrinkage and selection operator (LASSO) algorithms were employed step by step to select the optimal subset of radiomics features for predicting pT3a upstaging in the training set. In detail, the variance threshold method was first applied to obtain features with variance greater than the threshold (threshold = 0.75 in this study). Further, the select K best method was utilized to choose features with a *p*-value lower than 0.05, indicating a significant correlation with classification results. Lastly, LASSO, with 10-fold cross-testing, was adopted to select the most relevant feature dataset for predicting pT3a upstaging. The features obtained were then employed to construct the radiomics signature. The radiomics signature was calculated for each patient via a linear regression of the obtained features and weights according to their respective coefficients. The formula of the Rad_Score is as follows:

$$\mathrm{Rad}\_Score=Intercept+\sum_{i=1}^{n}\mathrm{coefficients}[i]\times Feature[i]$$

where *Intercept* is the Lasso regression intercept, *n* is the total number of features screened by the Lasso algorithm, coefficient[*i*] is the Lasso coefficient of the ith feature, and *Feature*[*i*] is the ith feature.

### 2.6. Model Construction and Evaluation

Univariate and multivariate logistic regression analyses were performed to select predictive clinical factors for pT3a upstaging in the training set. And the risk factors associating with pT3a upstaging were used to develop a clinical model.

The predictive performance of the radiomics signatures, clinical model, and nomogram was evaluated by AUC with 95% confidence intervals (CIs) in the training and testing set. The accuracy, sensitivity, specificity, precision, F1-score, positive predictive value (PPV), negative predictive value (NPV) were calculated for each model in both sets. The calibration curves were plotted to assess the goodness of fit of three models by using the Hosmer–Lemeshow test in both the training and the testing set. Decision curve analysis (DCA) was conducted to evaluate clinical practicability by quantifying the net benefits at different threshold probabilities.

### 2.7. Statistical Analysis

All statistical analyses were performed using R software (version 4.2.3) and Python (version 3.6.8). Categorical variables were compared using chi-square test 𝒳^2^ or Fisher’s exact test, expressed as frequency. Continuous variables were compared via t test or Mann–Whitney U test after a test of normality via Kolmogorov–Smirnov test, expressed as mean ± standard deviation. Delong’s test was used to compare AUC among different models. Two-tailed *p*-values lower than 0.05 were considered statistically significant.

## 3. Results

### 3.1. Clinical Data Analysis

A total of 169 cT1 patients with RCC were ultimately enrolled in this study, among which, 55 patients were diagnosed with pT3a upstaging (upstaging group) and 114 patients were diagnosed with consistent pT1 stage (non-upstaging group) after surgery. All baseline clinical factors of patients in the training and testing set are summarized in Table 1. Clinical factors, including age, sex, tumor size, RENAL score, BMI, Hb, NLR, AGR, and FIB, were equally balanced in the training and testing sets (*p* > 0.05). Significant differences were found in tumor size, RENAL score, and NLR between the upstaging and non-upstaging groups in both sets (*p* < 0.05).

### 3.2. Clinical Model Construction

Uni- and multivariable logistic regression analysis revealed that only tumor size (odds ratio (OR) 1.78; 95% confidence interval (CI) 1.15–2.75) and NLR (OR 1.62; 95% CI 1.21–2.18) were independent predictors for pT3a upstaging in cT1 RCC, and they were employed to develop a clinical model (Table 2).

### 3.3. Radiomics Feature Selection and Radiomics Signature Construction

**Figure 2:**
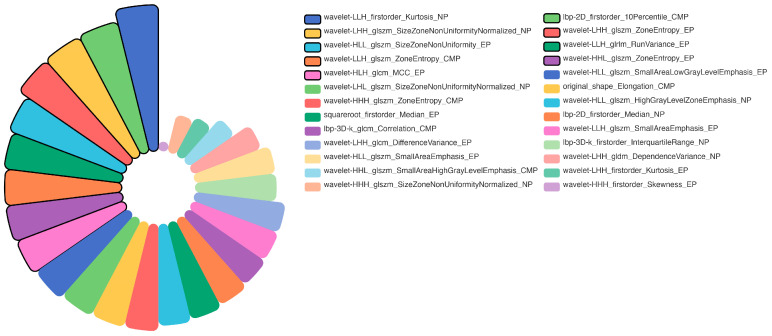
Twenty-six selected radiomics features and their respective contribution coefficients.

Overall, 1688 radiomics features were extracted from CMP, NP, and EP separately, and a total of 5064 features were extracted from each patient. Of these, 4128 features with ICC > 0.80 were entered into subsequent feature analysis. After a three-step procedure of feature selection, 6, 17, and 21 radiomics features with non-zero coefficients were finally selected for CMP, NP, and EP radiomics signature construction, respectively (Supplemental Table S1 and Figure S1). As for the rad-combined signature construction, 26 features were ultimately chosen, which include 6 features from CMP, 8 features from NP, and 12 from EP (Figure 2). The boxplots of the rad-combined signature in the training and testing sets are shown in the Supplemental Figure S2a,b.

### 3.4. Performance of Different Models

**Figure 3:**
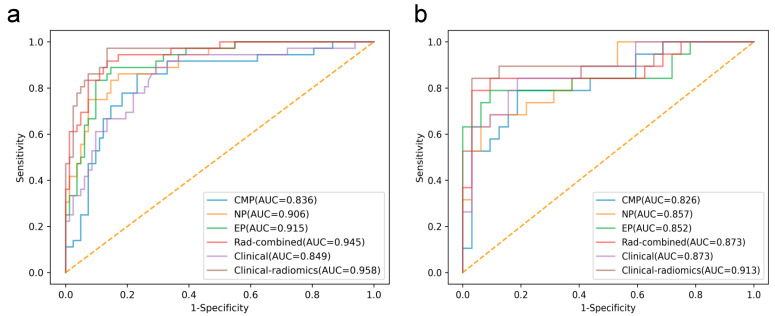
The receiving operating characteristics (ROC) curves of the radiomics signature, the clinical model, and clinical–radiomics combined model in the (**a**) training and (**b**) testing sets, respectively.

To assess the predictive performance of CECT-based radiomics models, we developed three separate radiomics signatures (CMP, NP, and EP, respectively) and a rad-combined signature (CMP + NP + EP). The AUCs of the aforementioned four models for predicting pT3a upstaging are presented in Table 3 and Figure 3. Among these models, the rad-combined signature exhibited good predictive performance with AUCs (95% CI) of 0.945 (0.904–0.986) and 0.873 (0.753–0.993) in the training and testing sets, respectively, outperforming three separate radiomics signature alone (Figure 3). Thus, the rad-combined signature was chosen as the final radiomics model for the nomogram construction.

By integrating the radiomics model with clinical risk factors, the combined clinical–radiomics model achieved improved predictive performance, with AUCs (95% CI) of 0.958 (0.921–0.995) and 0.913 (0.814–1.000) in the training and testing sets, respectively. The Delong test was performed to compare the AUCs of the radiomics model, the clinical model, and the combined clinical–radiomics model. There was significant difference between the clinical model and the clinical–radiomics model (*p* < 0.05) but not in the testing set. However, it is noteworthy that clinical factors plus the radiomics signature adds incremental value in improving the predictive performance.

### 3.5. Nomogram Construction and Testing

**Figure 4:**
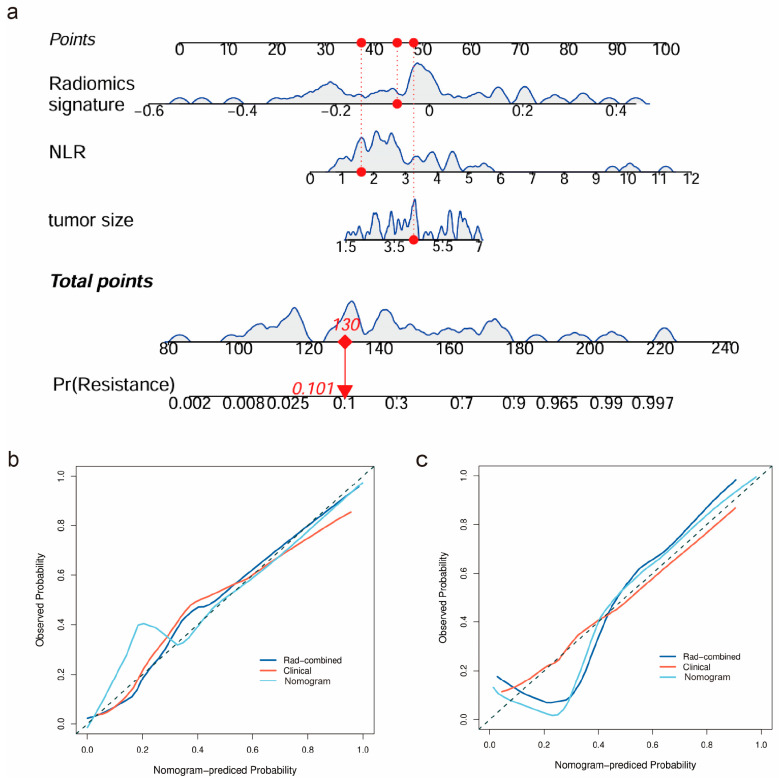
(**a**) The nomogram obtained by integrating the clinical risk factors and rad-combined signature. In the radiomics nomogram, one of the non-upstaging cT1 patients with RCC was presented as an example to illustrate the application of the nomogram. The distributions of the radiomics signature, NLR, and tumor size are shown on each scale. To utilize the nomogram, each variable of individual patients is positioned on the corresponding axis. The red dots and vertical red lines are drawn to the top point scale to determine the points assigned to each variable. The total points of this patient are 130, corresponding to a probability of 10% on the bottom axis for predicting pT3a upstaging. Calibration curves of the nomogram in the training (**b**) and testing (**c**) sets.

**Figure 5:**
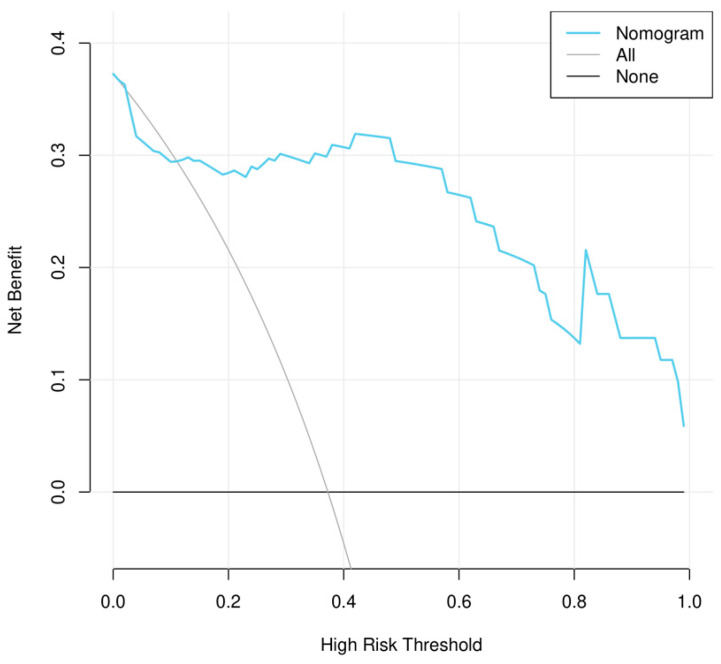
Decision curve analysis (DCA) for the nomogram in the testing set. The *y*-axis represents the net benefit, and the *x*-axis represents the threshold probability. In the current study, the threshold probability was 2%, and the corresponding net benefit is 0.378.

As the combined clinical–radiomics model exhibited the best predictive performance, a nomogram for the visualization of the combined model is shown in Figure 4a. The calibration curve in Figure 4b,c exhibits good concordance between the predicted and observed probability of pT3a upstaging in both the training and the testing sets (Brier score = 0.082 and 0.098), where a Brier score approaching 0 is better. The decision curve analysis (DCA) of the clinical model, the rad-combined model, and the clinical nomogram are shown in Figure 5. In our study, the threshold probability of the decision curve is 2%, and the corresponding net benefit is 0.378.

**Figure 6:**
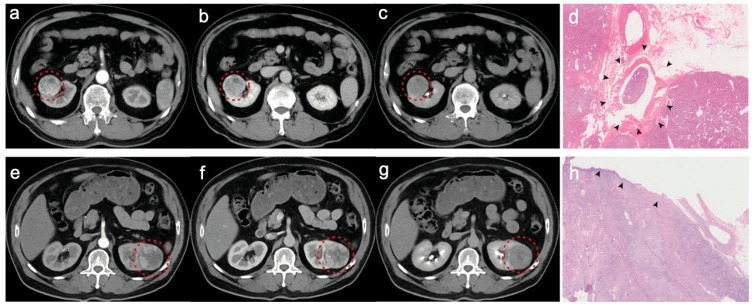
Representative CECT and pathologic images of two cases with upstaging (**a**–**d**) and non-upstaging RCC (**e**–**h**). CECT images (**a**–**c**) depicting a 61-year-old male patient presenting with a soft mass in the right kidney (red circles). The hematoxylin and eosin (HE)-stained image (**d**) revealed that the renal mass has invaded the renal vein, resulting in thrombosis (black arrows). CECT images (**e**–**g**) depicting a 58-year-old male patient presenting with a soft mass on the left kidney (red circles). The hematoxylin and eosin (HE)-stained image (**d**) revealed that the renal mass maintains a clear boundary (black arrows).

The representative CECT and pathologic images of two cases with upstaging and non-upstaging cT1 RCC are shown in Figure 6.

## 4. Discussion

In the current study, we primarily developed a nomogram to predict pT3a upstaging from localized cT1 RCC. The constructed nomogram integrated the radiomics signature based on CECT images with the clinical risk factors to improve the non-invasive and individualized prediction of tumor upstaging, outperforming both the radiomics signature and clinical factors alone. In addition, the calibration and DCA curves have demonstrated the good predictive efficiency and clinical usefulness of the nomogram.

Precision staging for RCC can be challenging in clinical practice because it usually grows as a spherical and well-defined mass with subtle invasion into the renal sinus, veins, or perinephric fat tissue. These microscopic invasions are often indistinguishable to the naked eye but can lead to occult T3a upstaging at final pathology [30]; whereas pathological upstaging poses a dilemma for clinical decision making. Increasing multi-institutional analysis revealed that patients with pT3a upstaging from cT1 RCC had higher relapse risk and cancer-specific deaths [3,9,16,31,32]. Therefore, the pre-identification of those cT1 patients who are at high risk of adverse pT3a upstaging is essential. It could assist in risk stratification prior to treatment and facilitate the selection of potential candidates for closer follow-up.

CECT is the standard modality for detecting and staging RCC. However, CT findings alone have insufficient sensitivity in detecting the subtle infiltration of perinephric fat [33]. Radiomics has demonstrated its efficacy as a valuable tool in the assessment and prognosis of RCC. A radiomics study indicated that the predictive model based on the logistic regression algorithm could efficiently predict the responses of patients with metastatic RCC (mRCC) to immunotherapy [34]. Accordingly, we hypothesized that CECT radiomics signatures might provide holistic information related to the tumor heterogeneity of RCC and facilitate the prediction of pathologic upstaging for patients with cT1 RCC.

In the current study, the CECT radiomics signature built in this study exhibited satisfactory performance in predicting pT3a upstaging, with an AUC of 0.873 in the testing set. Our results showed that the final radiomics signature incorporating “CMP + NP + EP” achieved superior predictive performance compared to any single-phase radiomics signature alone, suggesting that the multi-phase CECT scans offer a comprehensive analysis of tumor intensity, cellularity, and vascularization. Zhou et al. also asserted that the multi-phase-combined CECT radiomics model exhibited a better performance than single-phase radiomics model in distinguishing the Fuhrman grade of ccRCC [26]. In our study, a total of 26 radiomics features were ultimately selected using variance threshold, select K best, and LASSO methods to form the final radiomics signature. In the selected feature set, the wavelet-LLH_firstorder_Kurtosis of the NP has contributed to the greatest coefficient, indicating a positive correlation with pT3a upstaging. Wavelet transform-based radiomics features were obtained via the wavelet decomposition of original images, and they are able to capture the subtle textural variations across tumor volumes [35]. Kurtosis is a measure of the tailedness of the distribution of values in the image ROI. Other significant radiomics features selected for the radiomics signature were primarily high-order features derived from texture features, including GLSZM, GLCM, GLDM, and GLRLM. Several studies have demonstrated that the quantitative texture radiomics features, invisible to naked human eyes, has emerged as a valuable tool for evaluating heterogeneity and characterizing intratumor information [36,37]. As stated above, radiomics features have the potential to characterize tumor heterogeneity and facilitate the prediction of pT3a upstaging in cT1 RCC.

A recent study attempted to perform radiomics analysis based solely on CMP images to predict pT3a upstaging before surgery in individuals diagnosed with cT1b-2N0M0 RCC [38]. In their study, the best radiomics model using five extracted features yielded an AUC of 0.76, which is comparatively inferior to our own findings (AUC = 0.873). Furthermore, their study suffered from inherent limitations due to a lack of independent testing to provide reliable results on the predictive performance of the radiomics models. In contrast, the whole sample in our study was randomly split into training and testing sets, with each model being assessed in the testing set. Several studies have investigated predictive models consisting of clinical factors to predict pT3a upstaging [39,40]. Cao et al. developed a predictive model with preoperative blood indexes and oncological characteristics, including age, the ratio of the maximum and minimum tumor diameter (ROD), FIB, and tumor size [39]. Nevertheless, the final predictive model demonstrated a moderate performance, with an AUC of 0.712 in the validation set. In our study, we developed a nomogram with the advantage of integrating clinical risk factors, including tumor size and NLR, and achieved higher AUCs of 0.958 and 0.913 in both the training and the testing set, respectively. As the size of renal tumors increases, there exists a sharp probability of extrarenal and especially sinus invasion, particularly with respect to tumor sizes > 4 cm [41,42]. In addition, elevated NLR has been reported to be a significant negative predictor of oncological outcomes and cancer-specific survival in patients with RCC due to its promotion in the tumor [43,44,45]. Finally, the DCA demonstrated a favorable clinical net benefit of the nomogram.

To the best of our knowledge, limited research has been conducted to develop a model to predict pT3a upstaging stage at final pathology in cT1 patients with RCC by combining the radiomics signature and clinical risk factors. Integrating radiomic features with clinical risk factors significantly enhances predictive accuracy compared to using clinical factors alone. Pierre et al. were the first to develop a model using clinical features to predict the risk of pT3a stage in cT1 clear cell renal cell carcinoma [46]. Among the 236 patients who underwent partial or radical nephrectomy between 2005 and 2019, 25 patients (10.6%) were at the pT3a stage. A multivariable logistic regression model was fitted to predict pT3a using age (median age 69 vs. 62 years old, *p* < 0.05) and tumor size (median size 4.7 vs. 3.5 cm, *p* < 0.01). The accuracy of the model was 81% (95% CI: 71.8–89.6%). No differences were observed regarding sex, pole involvement, marginal location, surface features, renal sinus involvement, collecting system involvement, or exophytic rate. In our new model, significant differences were found in tumor size and NLR between the upstaging and the non-upstaging groups. There were no differences in gender, age, RENAL score, BMI, Hb, AGR, and FIB. The effect of tumor size on pathological upstaging was significantly different in our prediction model and Pierre’s prediction model. Moreover, 13 studies, including 21,869 patients, were identified in a systematic review and meta-analysis; the results showed that age, tumor size, and RENAL score were predictors of pathology upstaging, which is associated with overall survival (OS), cancer-specific survival, and progression-free survival (PFS) [47]. Therefore, in the preoperative preparation for renal cell carcinoma, patients with larger tumors should be given more consideration to improve their survival.

Our study has several limitations. First, this study was based on a single center and incorporated a relatively small dataset. To enhance the generalizability of radiomics models, further investigations should be explored and validated using larger multi-institutional datasets.

Second, the manual segmentation process of the entire tumor in three-phasic CECT was time-consuming and complicated; therefore, automated segmentation tools with accurate lesion identification should be developed in future studies.

## 5. Conclusions

The nomogram integrating the rad-combined signature with clinical risk factors could effectively predict pT3a upstaging in patients diagnosed with cT1 RCC prior to surgery. Thus, the nomogram might prove to be a reliable and non-invasive tool with which to support clinical decision-making, and it possesses great potential in the individualized prediction of pT3a upstaging in cT1 RCC.

## Figures and Tables

**Table 1:** Baseline patient characteristics in the training and testing set.

c	Training Cohort (*n* = 118)			Validation Cohort (*n* = 51)			pInter
	Upstaging * (*n* = 36)	Non-Upstaging * (n = 82)	pIntra	Upstaging (*n* = 19)	Non-Upstaging (*n* = 32)	pIntra	
Age(years), median [IQR]	65.50 [57.50, 73.25]	62.50 [54.25, 67.00]	0.143	64.47 (9.95)	59.00 (10.14)	0.067	0.71
Sex, no. (%)			0.764			0.82	1
Female	8 (22.2)	22 (26.8)		4 (21.1)	9 (28.1)		
Male	28 (77.8)	60 (73.2)		15 (78.9)	23 (71.9)		
Tumor size (cm), mean (SD)	5.06 (1.37)	3.94 (1.35)	<0.001	5.37 (1.20)	3.73 (1.34)	<0.001	0.76
RENAL score, median [IQR]	8.50 [7.00, 9.00]	6.00 [5.00, 8.00]	<0.001	9.00 [7.50, 10.00]	6.00 [5.00, 8.00]	0.001	0.33
BMI, median [IQR]	25.52 [23.65, 28.73]	25.98 [23.42, 27.70]	0.829	26.15 (3.09)	26.13 (3.06)	0.985	0.46
Hb, mean (SD)	134.81 (17.47)	138.35 (14.65)	0.256	127.05 (20.98)	142.16 (14.75)	0.004	0.79
NLR, median [IQR]	2.85 [1.93,6.26]	1.64 [1.27, 2.34]	<0.001	3.96 [2.68, 5.20]	2.17 [1.66, 2.74]	0.001	0
AGR, median [IQR]	1.70 [1.40, 1.80]	1.60 [1.50, 1.70]	0.418	1.47 (0.28)	1.59 (0.23)	0.107	0.15
FIB, median [IQR]	3.24 [2.78,3.55]	2.67 [2.34, 3.04]	<0.001	2.97 [2.66, 4.49]	2.53 [2.43, 2.90]	0.06	0.97

* Upstaging represents pT3a upstaging after surgery in patients with cT1 RCC, and non-upstaging represents a consistent pT1 stage in patients with cT1 RCC.

**Table 2:** Logistic regression analysis of clinical factors for their association with pT3a upstaging in the training cohort.

Clinic-Radiological Factors	Univariable Analysis		Multivariable Analysis	
	OR	*p*	OR	*p*
Sex (male vs. female)	1.28 (0.51–3.24)	0.6	NA	NA
Age (years)	1.03 (0.99–1.06)	0.12	NA	NA
Tumor size (cm)	1.82 (1.32–2.50)	<0.05	1.78 (1.15–2.75)	0.01
RENAL score	1.61 (1.24–2.08)	<0.05	1.19 (0.85–1.65)	0.31
BMI	0.99 (0.89–1.11)	0.92	NA	NA
Hb	0.99 (0.96–1.01)	0.26	NA	NA
NLR	1.68 (1.25–2.26)	<0.05	1.62 (1.21–2.18)	0
AGR	1.98 (0.39–10.13)	0.41	NA	NA
FIB	2.75 (1.46–5.18)	<0.05	1.73 (0.96–3.12)	0.07

NA: not applicable.

**Table 3:** Predictive performances of different models in the training and testing sets.

Models	Task	AUC (95% CI)	Accuracy	Sensitivity	Specificity	PPV	NPV	Precision	F1-Score
CMP	Training	0.836 (0.754–0.918)	0.771	0.861	0.732	0.585	0.923	0.585	0.697
	Testing	0.826 (0.704–0.948)	0.765	0.684	0.812	0.684	0.812	0.684	0.684
NP	Training	0.906 (0.850–0.962)	0.831	0.861	0.817	0.674	0.931	0.674	0.756
	Testing	0.857 (0.753–0.961)	0.824	0.684	0.906	0.812	0.829	0.812	0.743
EP	Training	0.915 (0.863–0.967)	0.856	0.861	0.854	0.721	0.933	0.721	0.785
	Testing	0.852 (0.722–0.982)	0.824	0.789	0.844	0.75	0.871	0.75	0.769
Rad-combined	Training	0.945 (0.904–0.986)	0.881	0.889	0.878	0.762	0.947	0.762	0.821
	Testing	0.873 (0.753–0.993)	0.882	0.737	0.969	0.933	0.861	0.933	0.824
Clinical	Training	0.849 (0.771–0.926)	0.763	0.722	0.78	0.591	0.865	0.591	0.65
	Testing	0.873 (0.771–0.976)	0.765	0.842	0.719	0.64	0.885	0.64	0.727
Clinical–radiomics	Training	0.958 (0.921–0.995)	0.89	0.889	0.89	0.78	0.948	0.78	0.831
	Testing	0.913 (0.814–1.000)	0.902	0.789	0.969	0.938	0.886	0.938	0.857

## Data Availability

The original contributions presented in this study are included in the article/supplementary material. Further inquiries can be directed to the corresponding author.

## Supplementary Materials

The following supporting information can be downloaded at https://www.mdpi.com/article/10.3390/diagnostics15040443/s1: Table S1: The results of feature selection from CMP, NP, and EP; Figure S1: Radiomics feature selection using LASSO algorithm; Figure S2: The box plots for rad-combined signature in training and testing datasets, categorized by upstaging and non-upstaging groups.

## Abbreviations

The following abbreviations are used in this manuscript:

RCC	Renal cell carcinoma
CECT	Contrast-enhanced CT
CMP	Corticomedullary phase
NP	Nephrographic phase
EP	Excretory phase
LASSO	Least absolute shrinkage and selection operator
ROC	Receiver operator characteristic
DCA	Decision curve analysis
NLR	Neutrophil–lymphocyte ratio
PN	Partial nephrectomy
RN	Radical nephrectomy
BMI	Body mass index
Hb	Hemoglobin
AGR	Albumin–globulin ratio
FIB	Fibrinogen

## References

[1] Motzer R.J., Jonasch E., Agarwal N., Alva A., Baine M., Beckermann K., Carlo M.I., Choueiri T.K., Costello B.A., Derweesh I.H. (2022). Kidney Cancer, Version 3.2022, NCCN Clinical Practice Guidelines in Oncology. J. Natl. Compr. Cancer Netw..

[2] Siegel R.L., Miller K.D., Wagle N.S., Jemal A. (2023). Cancer statistics, 2023. CA Cancer J. Clin..

[3] Ljungberg B., Albiges L., Abu-Ghanem Y., Bedke J., Capitanio U., Dabestani S., Fernandez-Pello S., Giles R.H., Hofmann F., Hora M. (2022). European Association of Urology Guidelines on Renal Cell Carcinoma: The 2022 Update. Eur. Urol..

[4] Thompson R.H., Siddiqui S., Lohse C.M., Leibovich B.C., Russo P., Blute M.L. (2009). Partial versus radical nephrectomy for 4 to 7 cm renal cortical tumors. J. Urol..

[5] Campbell S., Uzzo R.G., Allaf M.E., Bass E.B., Cadeddu J.A., Chang A., Clark P.E., Davis B.J., Derweesh I.H., Giambarresi L. (2017). Renal Mass and Localized Renal Cancer: AUA Guideline. J. Urol..

[6] Abou Elkassem A.M., Lo S.S., Gunn A.J., Shuch B.M., Dewitt-Foy M.E., Abouassaly R., Vaidya S.S., Clark J.I., Louie A.V., Siva S. (2021). Role of Imaging in Renal Cell Carcinoma: A Multidisciplinary Perspective. Radiographics.

[7] Cortellini A., Buti S., Bersanelli M., Cannita K., Pinterpe G., Venditti O., Verna L., Porzio G., Natoli C., Tinari N. (2020). Predictive Ability for Disease-Free Survival of the GRade, Age, Nodes, and Tumor (GRANT) Score in Patients with Resected Renal Cell Carcinoma. Curr. Urol..

[8] Li J., Zhang Y., Teng Z., Han Z. (2019). Partial nephrectomy versus radical nephrectomy for cT2 or greater renal tumors: A systematic review and meta-analysis. Minerva Urol. Nefrol..

[9] Ramaswamy K., Kheterpal E., Pham H., Mohan S., Stifelman M., Taneja S., Huang W.C. (2015). Significance of Pathologic T3a Upstaging in Clinical T1 Renal Masses Undergoing Nephrectomy. Clin. Genitourin. Cancer.

[10] Chevinsky M., Imnadze M., Sankin A., Winer A., Mano R., Jakubowski C., Mashni J., Sjoberg D.D., Chen Y.B., Tickoo S.K. (2015). Pathological Stage T3a Significantly Increases Disease Recurrence across All Tumor Sizes in Renal Cell Carcinoma. J. Urol..

[11] Shah P.H., Moreira D.M., Patel V.R., Gaunay G., George A.K., Alom M., Kozel Z., Yaskiv O., Hall S.J., Schwartz M.J. (2017). Partial Nephrectomy is Associated with Higher Risk of Relapse Compared with Radical Nephrectomy for Clinical Stage T1 Renal Cell Carcinoma Pathologically Up Staged to T3a. J. Urol..

[12] Srivastava A., Patel H.D., Joice G.A., Semerjian A., Gorin M.A., Johnson M.H., Allaf M.E., Pierorazio P.M. (2018). Incidence of T3a up-staging and survival after partial nephrectomy: Size-stratified rates and implications for prognosis. Urol. Oncol..

[13] Lee H., Lee M., Lee S.E., Byun S.S., Kim H.H., Kwak C., Hong S.K. (2018). Outcomes of pathologic stage T3a renal cell carcinoma up-staged from small renal tumor: Emphasis on partial nephrectomy. BMC Cancer.

[14] Ball M.W., Bezerra S.M., Gorin M.A., Cowan M., Pavlovich C.P., Pierorazio P.M., Netto G.J., Allaf M.E. (2015). Grade heterogeneity in small renal masses: Potential implications for renal mass biopsy. J. Urol..

[15] Fukui S., Miyake M., Iida K., Onishi K., Hori S., Morizawa Y., Kagebayashi Y., Fujimoto K. (2019). The Preoperative Predictive Factors for Pathological T3a Upstaging of Clinical T1 Renal Cell Carcinoma. Diagnostics.

[16] Gorin M.A., Ball M.W., Pierorazio P.M., Tanagho Y.S., Bhayani S.B., Kaouk J.H., Rogers C.G., Stifelman M.D., Khalifeh A., Kumar R. (2013). Outcomes and predictors of clinical T1 to pathological T3a tumor up-staging after robotic partial nephrectomy: A multi-institutional analysis. J. Urol..

[17] Xu P., Zhang S., Cao B., Li Y., Huang J., Lin W., Cheng J., Li H., Chen W., Zhu Y. (2022). Predictive value of renal tumor contour irregularity score in pathological T3a upstaging of clinical T1 renal cell carcinoma: A multi-institutional study. Urol. Oncol..

[18] Hotker A.M., Karlo C.A., Zheng J., Moskowitz C.S., Russo P., Hricak H., Akin O. (2016). Clear Cell Renal Cell Carcinoma: Associations Between CT Features and Patient Survival. AJR Am. J. Roentgenol..

[19] Elsayed Sharaf D., Shebel H., El-Diasty T., Osman Y., Khater S.M., Abdelhamid M., Abou El Atta H.M. (2022). Nomogram predictive model for differentiation between renal oncocytoma and chromophobe renal cell carcinoma at multi-phasic CT: A retrospective study. Clin. Radiol..

[20] Shebel H., Abou El Atta H.M., El-Diasty T., Sharaf D.E. (2024). Predictive quantitative multidetector computed tomography models for characterization of renal cell carcinoma subtypes and differentiation from renal oncocytoma: Nomogram algorithmic approach analysis. Egypt. J. Radiol. Nucl. Med..

[21] Teishima J., Hayashi T., Kitano H., Sadahide K., Sekino Y., Goto K., Inoue S., Honda Y., Sentani K., Awai K. (2020). Impact of radiological morphology of clinical T1 renal cell carcinoma on the prediction of upstaging to pathological T3. Jpn. J. Clin. Oncol..

[22] Gillies R.J., Kinahan P.E., Hricak H. (2016). Radiomics: Images Are More than Pictures, They Are Data. Radiology.

[23] Tomaszewski M.R., Gillies R.J. (2021). The Biological Meaning of Radiomic Features. Radiology.

[24] Demirjian N.L., Varghese B.A., Cen S.Y., Hwang D.H., Aron M., Siddiqui I., Fields B.K.K., Lei X., Yap F.Y., Rivas M. (2022). CT-based radiomics stratification of tumor grade and TNM stage of clear cell renal cell carcinoma. Eur. Radiol..

[25] Yang G., Nie P., Yan L., Zhang M., Wang Y., Zhao L., Li M., Xie F., Xie H., Li X. (2022). The radiomics-based tumor heterogeneity adds incremental value to the existing prognostic models for predicting outcome in localized clear cell renal cell carcinoma: A multicenter study. Eur. J. Nucl. Med. Mol. Imaging.

[26] Zhou Z., Qian X., Hu J., Geng C., Zhang Y., Dou X., Che T., Zhu J., Dai Y. (2023). Multi-phase-combined CECT radiomics models for Fuhrman grade prediction of clear cell renal cell carcinoma. Front. Oncol..

[27] Gao R., Pang J., Lin P., Wen R., Wen D., Liang Y., Ma Z., Liang L., He Y., Yang H. (2024). Identification of clear cell renal cell carcinoma subtypes by integrating radiomics and transcriptomics. Heliyon.

[28] Rallis K.S., Kleeman S.O., Grant M., Ordidge K.L., Sahdev A., Powles T. (2021). Radiomics for Renal Cell Carcinoma: Predicting Outcomes from Immunotherapy and Targeted Therapies-A Narrative Review. Eur. Urol. Focus.

[29] Elkassem A.A., Allen B.C., Sharbidre K.G., Rais-Bahrami S., Smith A.D. (2021). Update on the Role of Imaging in Clinical Staging and Restaging of Renal Cell Carcinoma Based on the AJCC 8th Edition, From the AJR Special Series on Cancer Staging. AJR Am. J. Roentgenol..

[30] Williamson S.R., Rao P., Hes O., Epstein J.I., Smith S.C., Picken M.M., Zhou M., Tretiakova M.S., Tickoo S.K., Chen Y.B. (2018). Challenges in Pathologic Staging of Renal Cell Carcinoma: A Study of Interobserver Variability Among Urologic Pathologists. Am. J. Surg. Pathol..

[31] Roberts W.W., Bhayani S.B., Allaf M.E., Chan T.Y., Kavoussi L.R., Jarrett T.W. (2005). Pathological stage does not alter the prognosis for renal lesions determined to be stage T1 by computerized tomography. J. Urol..

[32] Nayak J.G., Patel P., Saarela O., Liu Z., Kapoor A., Finelli A., Tanguay S., Rendon R., Moore R., Black P.C. (2016). Pathological Upstaging of Clinical T1 to Pathological T3a Renal Cell Carcinoma: A Multi-institutional Analysis of Short-term Outcomes. Urology.

[33] Liu Y., Song T., Huang Z., Zhang S., Li Y. (2012). The accuracy of multidetector Computed Tomography for preoperative staging of renal cell carcinoma. Int. Braz. J. Urol..

[34] Khene Z.E., Mathieu R., Peyronnet B., Kokorian R., Gasmi A., Khene F., Rioux-Leclercq N., Kammerer-Jacquet S.F., Shariat S., Laguerre B. (2021). Radiomics can predict tumour response in patients treated with Nivolumab for a metastatic renal cell carcinoma: An artificial intelligence concept. World J. Urol..

[35] Sudarshan V.K., Mookiah M.R., Acharya U.R., Chandran V., Molinari F., Fujita H., Ng K.H. (2016). Application of wavelet techniques for cancer diagnosis using ultrasound images: A Review. Comput. Biol. Med..

[36] Erdim C., Yardimci A.H., Bektas C.T., Kocak B., Koca S.B., Demir H., Kilickesmez O. (2020). Prediction of Benign and Malignant Solid Renal Masses: Machine Learning-Based CT Texture Analysis. Acad. Radiol..

[37] Varghese B.A., Chen F., Hwang D.H., Cen S.Y., Desai B., Gill I.S., Duddalwar V.A. (2018). Differentiation of Predominantly Solid Enhancing Lipid-Poor Renal Cell Masses by Use of Contrast-Enhanced CT: Evaluating the Role of Texture in Tumor Subtyping. AJR Am. J. Roentgenol..

[38] Shimada W., Kimura K., Tanaka H., Fukuda S., Yoshida S., Fujii Y. (2022). Significance of tumor shape irregularity: Radiomics analysis based on dynamic computed tomography for predicting pT3a upstaging in cT1b-2N0M 0 renal cell carcinoma. Int. J. Urol..

[39] Cao C., Kang X., Shang B., Shou J., Shi H., Jiang W., Xie R., Zhang J., Zhang L., Zheng S. (2022). A novel nomogram can predict pathological T3a upstaged from clinical T1a in localized renal cell carcinoma. Int. Braz. J. Urol..

[40] Liu H., Wang Z., Peng E., Chen Z., Tang K., Xia D. (2021). Added Value of Systemic Inflammation Markers in Predicting Clinical Stage T1 Renal Cell Carcinoma Pathologically Upstaged to T3a. Front. Oncol..

[41] Bonsib S.M. (2004). The renal sinus is the principal invasive pathway: A prospective study of 100 renal cell carcinomas. Am. J. Surg. Pathol..

[42] Williamson S.R., Taneja K., Cheng L. (2019). Renal cell carcinoma staging: Pitfalls, challenges, and updates. Histopathology.

[43] Asif A., Chan V.W., Osman F.H., Koe J.S., Ng A., Burton O.E., Cartledge J., Kimuli M., Vasudev N., Ralph C. (2023). The Prognostic Value of Neutrophil-to-Lymphocyte Ratio and Platelet-to-Lymphocyte Ratio for Small Renal Cell Carcinomas after Image-Guided Cryoablation or Radio-Frequency Ablation. Cancers.

[44] Pichler M., Hutterer G., Stoeckigt C., Chromecki T., Stojakovic T., Golbeck S., Eberhard K., Gerger A., Mannweiler S., Pummer K. (2013). Validation of the pre-treatment neutrophil–lymphocyte ratio as a prognostic factor in a large European cohort of renal cell carcinoma patients. Br. J. Cancer.

[45] Kim J., Park J.S., Heo J.E., Elghiaty A., Jang W.S., Rha K.H., Choi Y.D., Ham W.S. (2019). Neutrophil-to-Lymphocyte Ratio Predicts Pathological Renal Sinus Fat Invasion in Renal Cell Carcinomas of </=7 cm with Presumed Renal Sinus Fat Invasion. Yonsei Med. J..

[46] Nocera L., Stolzenbach L.F., Ruvolo C.C., Wenzel M., Tian Z., Rosiello G., Bravi C.A., Candela L., Basile G., Larcher A. (2021). Predicting the risk of pT3a stage in cT1 clear cell renal cell carcinoma. Eur. J. Surg. Oncol..

[47] Veccia A., Falagario U., Martini A., Marchioni M., Antonelli A., Simeone C., Cormio L., Capitanio U., Mir M.C., Derweesh I. (2021). Upstaging to pT3a in Patients Undergoing Partial or Radical Nephrectomy for cT1 Renal Tumors: A Systematic Review and Meta-analysis of Outcomes and Predictive Factors. Eur. Urol. Focus.

