# Supplementary material for: Contrast-Enhanced CT-Based Radiomics Nomogram for Prediction of Pathologic T3a Upstaging in Clinical T1 RCC
Source: Diagnostics (Basel). 2025 Feb 12;15(4):443. doi: 10.3390/diagnostics15040443 (PMC11854503; doi:10.3390/diagnostics15040443)
Supplement: Supplementary file 1 [file diagnostics-15-00443-s001.zip › diagnostics-3426004-supplementary.pdf]

Supplementary Table S1: The results of feature selection from CMP, NP, and EP

CT sequences	Feature number	Individual features
CMP	6	original_shape_Maximum2DDiameterSlice<br>original_shape_MajorAxisLength<br>lbp-2D_firstorder_10Percentile<br>wavelet-LLH_glszm_ZoneEntropy<br>wavelet-HHH_glszm_ZoneEntropy<br>lbp-2D_firstorder_Median
NP	17	original_shape_Maximum2DDiameterSlice<br>wavelet-LLH_firstorder_Kurtosis<br>lbp-3D-m1_firstorder_Mean<br>lbp-2D_firstorder_Median<br>wavelet-LHH_glszm_SizeZoneNonUniformityNormalized<br>original_shape_Elongation<br>lbp-3D-k_firstorder_RobustMeanAbsoluteDeviation<br>lbp-3D-k_firstorder_InterquartileRange<br>wavelet-LLH_firstorder_90Percentile<br>wavelet-LHH_gldm_DependenceVariance<br>wavelet-LHH_firstorder_Skewness<br>wavelet-LHH_glcm_Imc2<br>wavelet-HLL_glszm_GrayLevelVariance<br>wavelet-HLH_glszm_SmallAreaEmphasis<br>wavelet-LHL_glszm_SizeZoneNonUniformityNormalized<br>wavelet-HLL_glszm_HighGrayLevelZoneEmphasis<br>wavelet-HHH_glszm_SizeZoneNonUniformityNormalized
EP	21	wavelet-HLL_glszm_SizeZoneNonUniformity<br>wavelet-LHH_glszm_ZoneEntropy<br>original_shape_Maximum2DDiameterSlice<br>original_shape_Sphericity<br>wavelet-HLL_glszm_SmallAreaLowGrayLevelEmphasis<br>lbp-3D-m2_firstorder_Maximum<br>wavelet-LLH_firstorder_10Percentile<br>wavelet-HHL_glszm_ZoneEntropy<br>wavelet-HLL_glszm_SmallAreaEmphasis<br>lbp-2D_firstorder_InterquartileRange<br>wavelet-LHH_glcm_DifferenceVariance<br>wavelet-HHH_firstorder_Skewness<br>wavelet-LHL_firstorder_Minimum<br>wavelet-LLH_glrlm_RunVariance<br>wavelet-HLL_firstorder_Mean<br>wavelet-LHH_firstorder_Kurtosis<br>squareroot_firstorder_Median<br>wavelet-LLH_glszm_SmallAreaEmphasis<br>wavelet-HLL_firstorder_Kurtosis<br>wavelet-LLH_ngtdm_Busyness<br>wavelet-HLH_glcm_MCC

After a three-step procedure of feature selection, 6, 17, and 21 radiomics features were selected from CMP, P, and EP images. The selected radiomics features are listed as above.

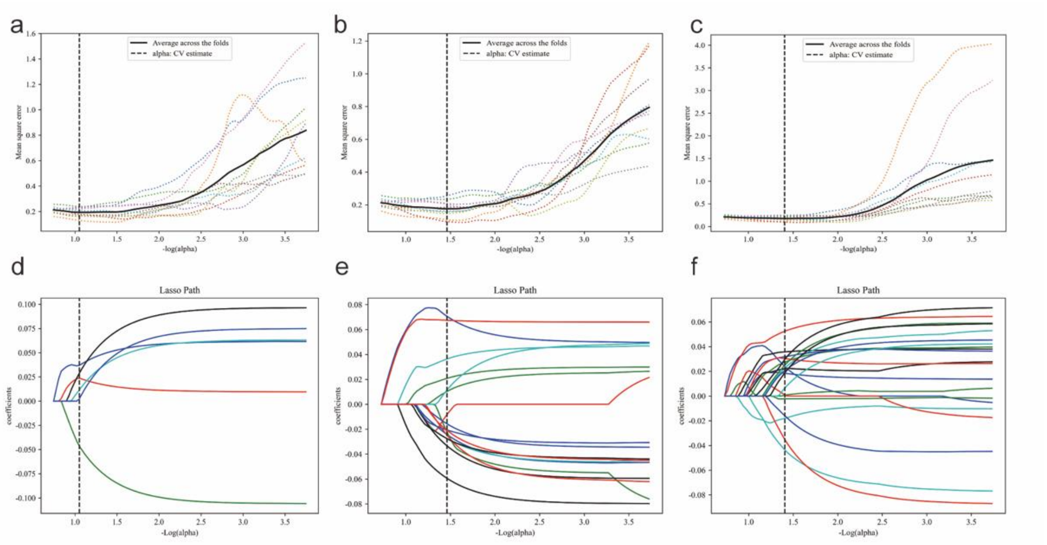

Supplementary Figure S1: Radiomics feature selection using LASSO algorithm. Identification of the optimal regulation parameter lambda (λ) in LASSO using 10-fold cross-validation for CMP (a), NP (b) and EP (c) images. LASSO coefficient profiles of the 6 radiomics features for CMP images (d), 17 radiomics features for NP images (e), and 21 radiomics features for EP images (f).

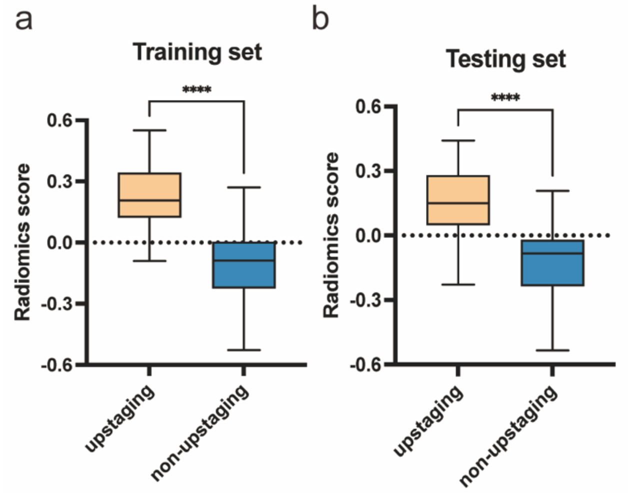

Supplementary Figure S2: The box plots for rad-combined signature in training and testing datasets, categorized by upstaging and non-upstaging groups.
